# Parallel Metabolomic Profiling of Cerebrospinal Fluid, Plasma, and Spinal Cord to Identify Biomarkers for Spinal Cord Injury

**DOI:** 10.1007/s12031-021-01903-w

**Published:** 2021-09-09

**Authors:** Hua Yang, Pengwei Zhang, Min Xie, Jianxian Luo, Jing Zhang, Guowei Zhang, Yang Wang, Hongsheng Lin, Zhisheng Ji

**Affiliations:** 1 Department of Orthopedics, The First Affiliated Hospital of Jinan University, No.601 West Huangpu Avenue, Tianhe, Guangzhou, 510630 China; 2 MOE Key Laboratory of Tumor Molecular Biology and Key Laboratory of Functional Protein Research of Guangdong Higher Education Institutes, College of Life Science and Technology, Institute of Life and Health Engineering, Jinan University, Guangzhou, 510632 China

**Keywords:** Metabolomics, Spinal cord injury, Biomarkers

## Abstract

Loss of physical and emotional health due to spinal cord injury (SCI) has been rapidly increasing worldwide. Effective evaluation of the severity of SCI is crucial to its prognosis. Herein, we constructed rat models of SCI with four different degrees of injury (sham group, light injury group, moderate injury group, and heavy injury group), using the surgical approach. Cerebrospinal fluid (CSF), plasma, and spinal cord were sampled at the sub-acute spinal cord (72 h post-injury) from each rat. The LC–MS-based metabolic profiling of these samples was performed according to a universal metabolome standard (UMS). The results demonstrated that 130, 104, and 128 metabolites were significantly altered within the CSF, plasma, and spinal cord samples, respectively. Among them, there were four differential metabolites, including uric acid, phosphorycholine, pyridoxine, and guanidoacetic acid, which were commonly identified within the CSF, plasma, and spinal cord samples. Further pathway analysis of these differential metabolites demonstrated a disturbance in the metabolism of glyoxylate and dicarboxylate and glycine, serine, and threonine which were associated with pathophysiologic consequence of spinal cord injury. In particular, phosphorycholine, pyridoxine, and guanidoacetic acid demonstrated a relationship with SCI severity. Thus, they could be utilized as potential metabolite biomarkers for SCI severity assessment.

## Introduction

There is a significant rise in the occurrence of spinal cord injury (SCI) each year due to the fast development of transport, mining, sports, and construction sectors (Singh et al. 2014). The impact of this type of an injury has lasting effects on the health and quality of life of an individual (Chan et al. 2016). Therefore, mechanism of SCI is one of the research hotspots (Alizadeh et al. 2019). However, the mechanisms regarding the transient release series of inflammatory (Wu et al. 2019a), biochemical self-destruct factors (i.e., calcium overload, free radical and excitatory amino acids, monoamine, neuropeptide, platelet activating factor) (Alizadeh et al. 2019; Hill et al. 2016), neuronal necrosis and apoptosis (Callizot et al. 2019), and initiation of endogenous repair factors after spinal cord injury remain unclear (Alizadeh et al. 2019). Due to the complexity of SCIs, traditional research has been difficult. Therefore, the emergence of metabolomics can help bring a new impetus to the study of SCI (Fujieda et al. 2012).

SCI has been evaluated clinically for quantification of neurologic impairment, as well as level of injury, which has impeded the validity for further efforts in order to intervene in the SCI setting (Wu et al. 2016). Novel treatments for spinal cord injury can be evaluated based on biomarkers, which are able to characterize the severity of injury, and correctly predict neurologic recovery (Elizei and Kwon 2017). Furthermore, SCI sparks pathophysiologic mechanisms, which is crucial for the discovery of novel SCI therapies. When the spinal cord becomes traumatized, it can cause disturbance of the whole metabolic cascade, which encompasses lipid and amino acid metabolism, as well as glycolysis and oxidative stress (Dulin et al. 2013). High throughput global profiling of the pool of metabolites in any biological system is performed, and metabolite candidates can be identified as potential biomarkers in the constantly evolving field of metabolomics. While prior studies have reported the metabolite changes after SCI (Dulin et al. 2013; Peng et al. 2014; Jiang et al. 2010), a global profiling of the metabolic network as a feedback to different degrees of subacute SCI has remained previously unreported.

Herein, we describe the parallel metabolomic profiling of the cerebrospinal fluid (CSF), plasma, and spinal cord from subacute SCI. Changes in metabolites of the CSF, plasma, and spinal cord in respect to different degrees of injury were identified in order to assess potential biomarkers for SCI. With LC–MS, the dataset demonstrated that metabolism of glyoxylate and dicarboxylate, glycine, serine, and threonine metabolism were related to pathophysiologic consequences of SCI. In addition, levels of uric acid, phosphorycholine, pyridoxine, and guanidoacetic acid were significantly altered in the spinal cord, CSF, and plasma samples, which can be prognostic biomarkers for prediction of SCI.

## Results

### Metabolomic Profiling of Spinal Cord, CSF and Plasma from Control and Spinal Cord Injured Rat

**Fig. 1:**
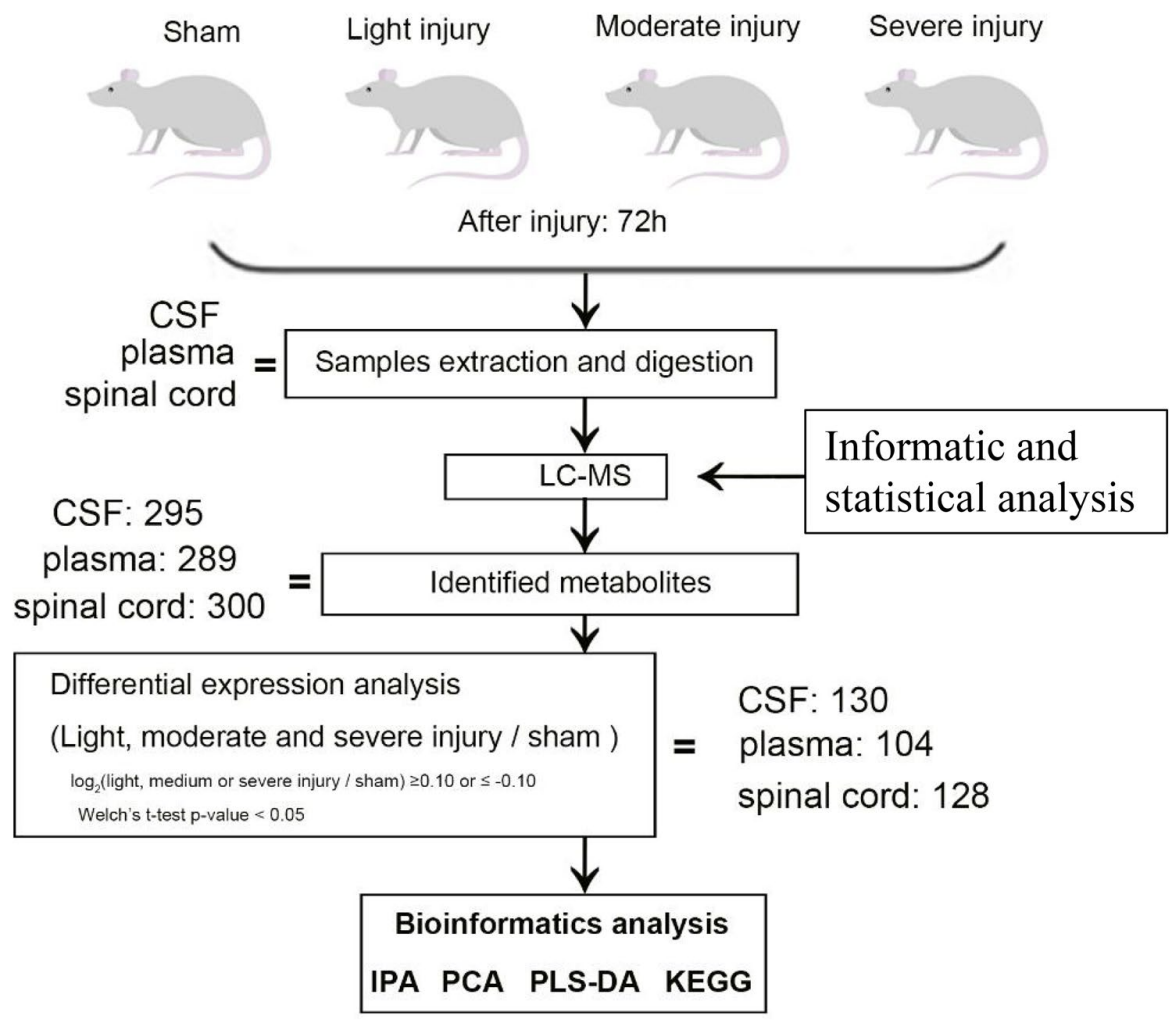
Workflow of UHPLC-HRMS-based untargeted metabolomic profiling of CSF, plasma and spinal cord tissue samples from control and spinal cord injured rats

**Fig. 2:**
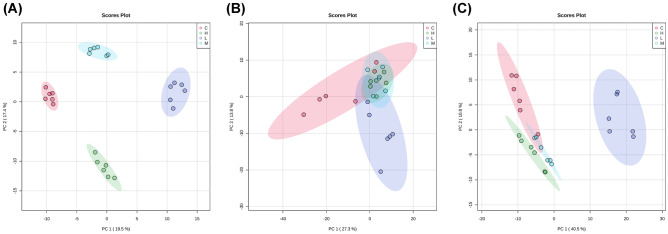
Principal component variable discriminant plots (PC 1 vs. PC 2) derived from LC–MS data between control and spinal cord injured groups. **A** CSF samples, explained variance 37.0%, **B** plasma samples, explained variance 41.1%, and **C** spinal cord tissue samples, explained variance 51.3%

Samples from rat spinal cord, CSF, and plasma were analyzed under the same metabolome analysis platform (Fig. 1). The metabolome platform was able to confidently identify 300, 289, and 295 metabolites in spinal cord, plasma, and CSF sample, respectively. The identified metabolites were then subjected to principal component analysis (PCA) in order to assess the effects of SCI (Fig. 2). PCA using the CSF samples showed the best cluster results (Fig. 2). Although the cluster effect is not as good as the CSF samples, PCS using plasma and spinal cord tissue samples were able to differentiate the SCI groups from controls. Thus, SCI triggered significant changes in the metabolomic profile of CSF, plasma, and spinal cord tissue.

### Selection of Metabolites Associated with the Spinal Cord Injury

**Fig. 3:**
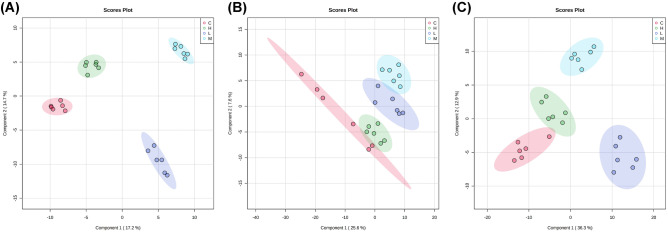
Supervised partial least squares-discriminant analysis (PLS-DA) using LC–MS data of identified metabolites in each sample class. The score plots show comparative metabolomics of control (C), light spinal cord injury (L), moderate injury (M) and heavy injury (H). **A** for CSF samples, **B** for plasma samples, and **C** for spinal cord tissue samples

**Fig. 4:**
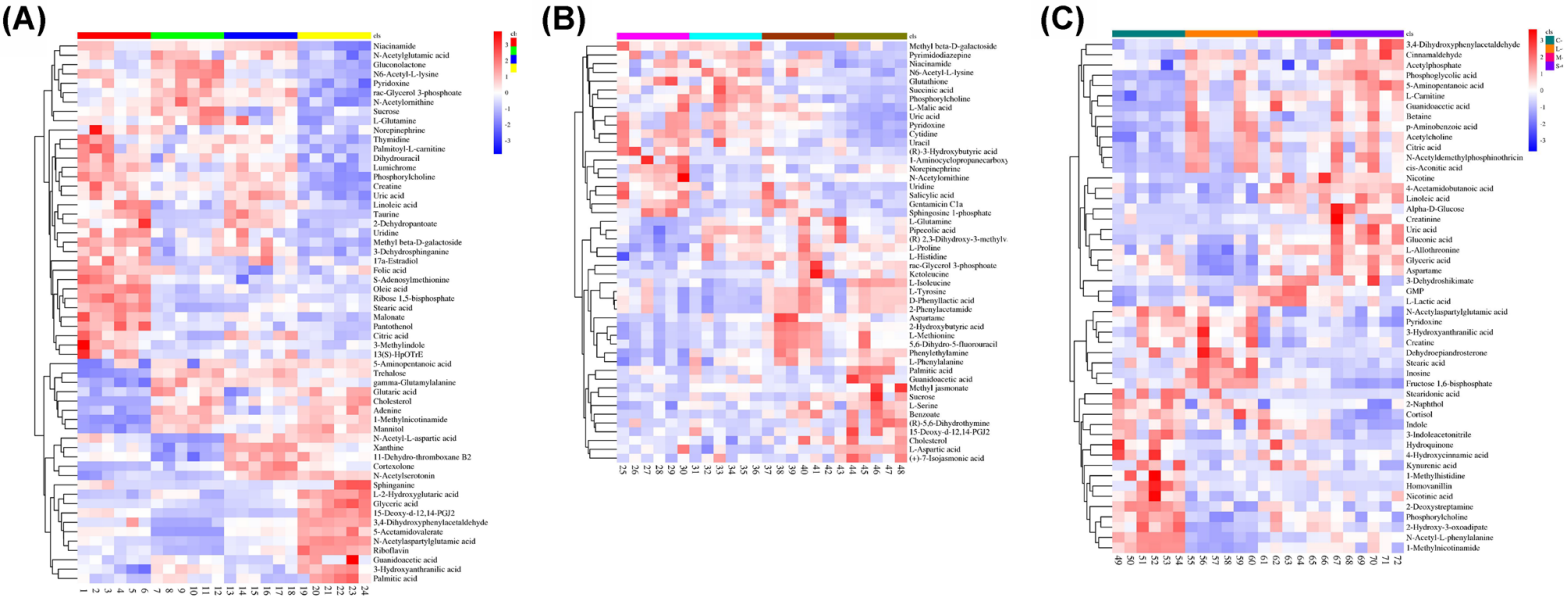
Heatmap analysis of metabolite levels with significant difference between control and spinal cord injured rats (one-way ANOVA) in **A** CSF samples, **B** plasma samples, **C** spinal cord samples. The row exhibits metabolite while the column signifies the samples. The level of significantly increased metabolites were in red, while the level of metabolites significantly decreased were shown in blue

In order to investigate the specific metabolites that are associated with SCI, the supervised multivariate analysis PLS-DA, as well as univariate analysis one-way ANOVA, was carried out. The PLS-DA model was able to appropriately categorize all samples across different groups (Fig. 3). Based on the results from a one-way ANOVA test, 130, 104, and 128 metabolites were found to be significantly altered between control and the SCI groups in the CSF, plasma, and spinal cord samples, respectively. The criteria utilized for selection of the metabolites that were associated with SCI are as follows: (i) a VIP value > 1 in PLS-DA and (ii) statistically significant changes in levels of metabolites between the control and spinal cord injured groups (one-way ANOVA test; *P* < 0.05). Under these criteria, 50 metabolites were identified using the spinal cord tissue samples, which indicated that these metabolites carried out a vital function in SCI. In addition, 48 metabolites were identified in the plasma samples and 46 in the CSF samples. In Fig. 4, the heatmap highlights the differential metabolites between the control and SCI groups. These metabolites were confidently determined based on their accurate mass and MS/MS fragmentation pattern.

### Common Significantly Altered Metabolites Among Spinal Cord, CSF, and Plasma Samples

**Fig. 5:**
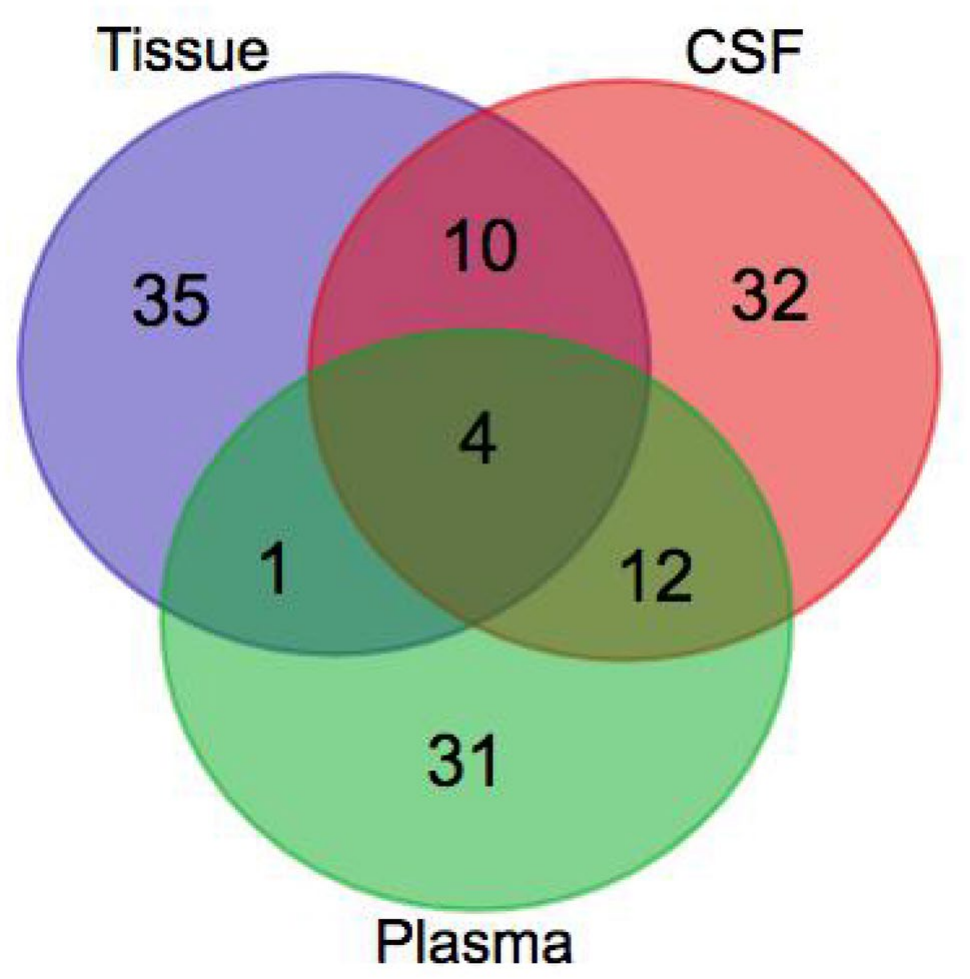
Venn diagram representing the number of common and specific differential metabolites in different sample classes

**Fig. 6:**
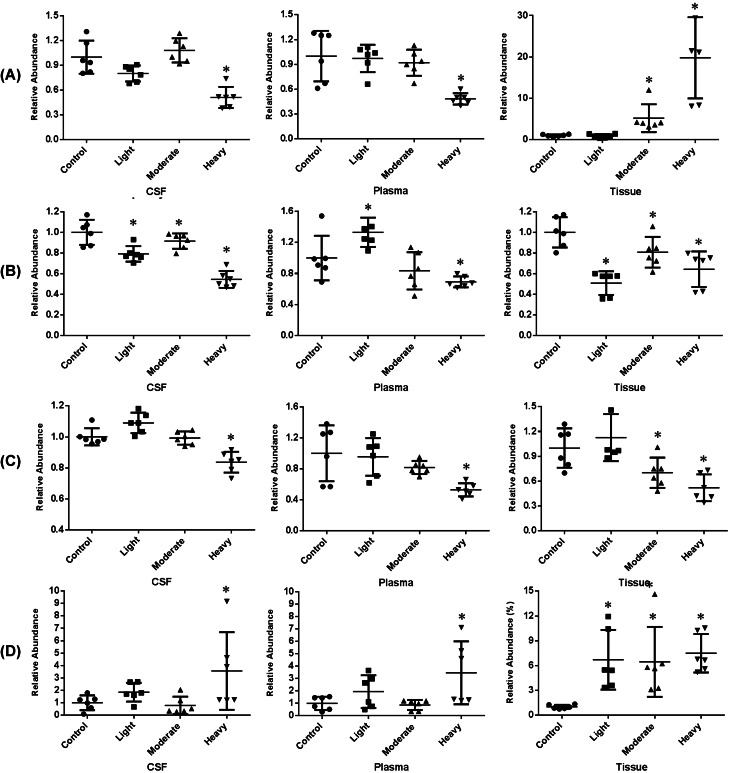
Metabolites showing significant differences between control versus spinal cord injured groups. Data are shown as scatter plots with individual point corresponding to each sample. **A** uric acid, **B** phosphorycholine, **C** pyridoxine, **D** guanidoacetic acid. Asterisk denotes *P* < 0.05 by unpaired student’s *t* test

The identified metabolites from the three cohorts were compared. While the spinal cord and CSF exhibited 14 common significantly changed metabolites (Table 1 and Fig. 5), the spinal cord and plasma only had five common metabolites that were significantly altered. Furthermore, four metabolites were shared between the spinal cord, CSF, and plasma samples. Interestingly, compared to the spinal cord samples, uric acid demonstrated the reverse trend in the plasma and CSF samples (Fig. 6).

**Table 1:** Fourteen common significantly altered metabolites in the CSF, plasma, and spinal cord injured tissue

No	*m/z*	Precursor ion type	rt	Mass error (ppm)	Chemical formula	KEGG ID	Metabolite name
1	191.0192	[M–H]^–^	82.19162	1.2	C_6_H_8_O_7_	C00158	Citric acid
2	105.0178	[M–H]^−^	86.21925	3.2	C_3_H_6_O_4_	C00258	Glyceric acid
3	130.0608	[M–H]^−^	101.7751	1.6	C_4_H_9_N_3_O_2_	C00300	Creatine
4	115.919	[M–H]^−^	874.9036	5.5	C_5_H_11_NO_2_	C00431	5-aminopentanoic acid
5	154.0585	[M + H]^+^	99.84333	0.5	C_7_H_7_NO_3_	C00632	3-hydroxyanthranilic acid
6	284.1852	[M]^+^	443.6896	0.8	C_18_H_36_O_2_	C01530	Stearic acid
7	281.2468	[M + H]^+^	872.8783	2.4	C_18_H_32_O_2_	C01595	Linoleic acid
8	137.0708	[M + H]^+^	92.65175	0.0	C_7_H_9_N_2_O	C02918	1-methylnicotinamide
9	152.0557	[M]^+^	340.9878	5.9	C_8_H_8_O_3_	C04043	3,4-dihydroxyphenylacetaldehyde
10	305.0977	[M + H]^+^	234.0309	0.0	C_11_H_16_N_2_O_8_	C12270	*N*-acetylaspartylglutamic acid
11	118.0612	[M + H]^+^	93.51683	0.7	C_3_H_7_N_3_O_2_	C00581	Guanidoacetic acid
12	184.0725	[M + H]^+^	91.65588	2.2	C_5_H_15_NO_4_P	C00588	Phosphorylcholine
13	169.0355	[M + H]^+^	131.2458	0.7	C_5_H_4_N_4_O_3_	C00366	Uric acid
14	170.0324	[M + H]^+^	99.62296	11.2	C_8_H_11_NO_3_	C00314	Pyridoxine

### Pathway Impact Analysis

**Fig. 7:**
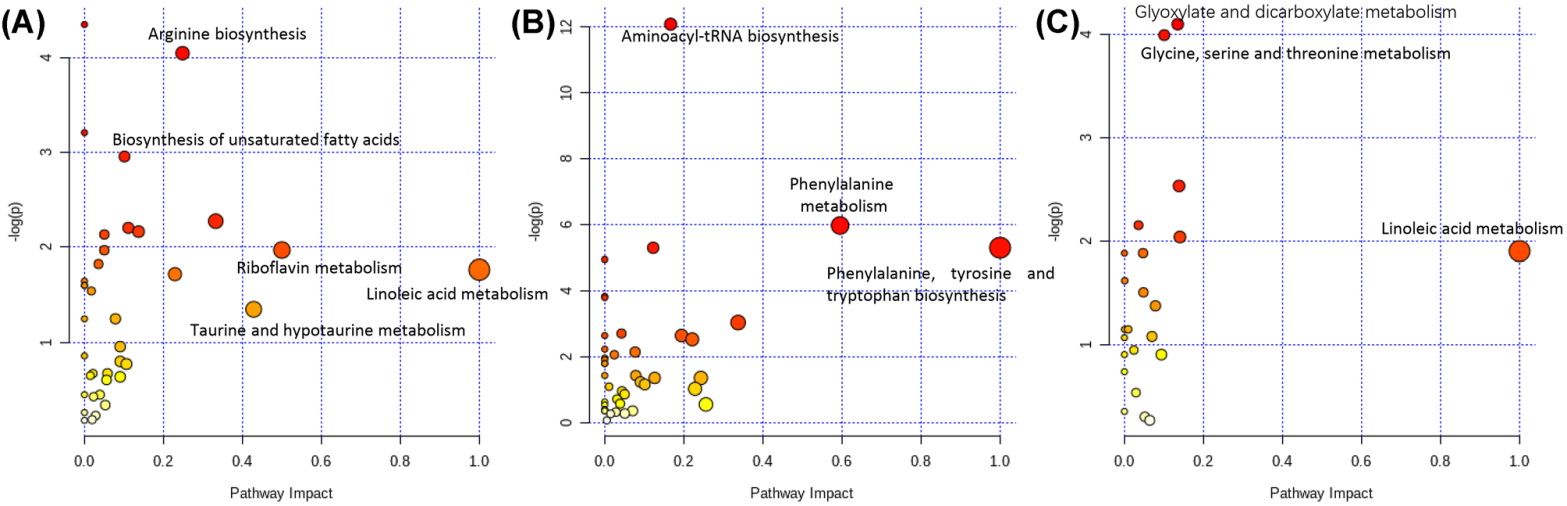
Pathway-impact analysis showing the metabolic pathways perturbed by spinal cord injury in **A** CSF **B** plasma, and **C** spinal cord tissue

The differentially identified metabolites from the different sample types were submitted to KEGG pathway analysis (Fig. 7). The threshold for significantly altered pathways was set at *P* < 0.05, using the hypogemeric test. Within the CSF, arginine biosynthesis, alanine, aspartate, and glutamate metabolism and biosynthesis of unsaturated fatty acids pathways were significantly altered, with *P* = 0.01, 0.02, and 0.04, respectively. Within the plasma and spinal cord tissue, eight and two metabolic pathways were significantly altered, respectively. Among them, the glyoxylate and dicarboxylate metabolic pathway in the spinal cord tissue was most relevant to the SCI response with *P* = 0.017. Within the plasma, pathways that are associated with amino acid and protein synthesis were affected.

## Discussion

Herein, we evaluated metabolic changes of the CSF, plasma, and spinal cord tissue, with respect to SCI. To the best of our best knowledge, this is the first study to investigate metabolite alterations in response to SCI through the use of parallel metabolomic profiling of the CSF, plasma, and spinal cord tissue. The aim of this study is to characterize metabolite changes in these samples, in response to spinal cord injury. Both plasma and CSF samples are an ideal source for the discovery of altered metabolites, which are able to reflect the injury responses within the spinal cord (Fujieda et al. 2012; Wu et al. 2016; Peng et al. 2014). However, metabolomic changes in injured tissue of the spinal cord have not yet been profiled. In order to better understand the parthenogenesis of the injured spinal cord, we measured metabolic changes of the injured spinal cord tissue directly. By comparing to the healthy controls, 50 metabolites were found to be significantly altered in response to the spinal cord injured tissue sample. Subsequently, pathway analysis of these differentiated metabolites demonstrated that alterations in the metabolism of glyoxylate and dicarboxylate, glycine, serine, and threonine metabolism were induced, which suggests that these pathways are related to the pathophysiologic consequence of SCI. This is the first report to link glycine, serine, and threonine metabolism with SCI.

We focused on the establishment of neurochemical biomarkers of SCI, utilizing either blood, CSF, or spinal cord tissue. CSF is intuitively more representative of parenchymal injury due to its proximity to the spinal cord and has been utilized in the investigation of biomarkers for additional neurologic conditions, which includes traumatic brain injury, stroke, and other neurodegenerative disorders (Elizei and Kwon 2017). Herein, we identified 58 differential metabolites between the control and spinal cord injured groups were selected from the CSF samples. Among them, 14 metabolites were also observed within the spinal cord injured tissue samples. Enrichment analysis demonstrated that these 14 metabolites were responsible for metabolism of glyoxylate and dicarboxylate, glycine, serine and threonine, arginine and proline, and biosynthesis of unsaturated fatty acids. It has been reported that CSF has a similar metabolome to plasma samples (Crews et al. 2009). We discovered 16 common differential metabolites within the plasma and CSF, which is significantly higher than a previous study using plasma and CSF. When evaluating the CSF concentrations at 24 h post-injury, we discovered that these proteins were distinctly expressed between AIS (American Spinal Injury Association) grades. Thus, they can be utilized in an ordinal logistic regression model in order to classify the AIS grade with an accuracy of 89%. They could also be utilized in the cervical spinal cord injury patients in order to predict segmental motor recovery with better accuracy than utilizing the baseline AIS grade (Elizei and Kwon 2017).

Due to the unavailability of spinal cord tissue in clinic, we aimed to identify significant neurochemical biomarkers that can reflect the process of SCI. Our results demonstrated that CSF samples shared more common differentially expressed metabolites (14 metabolites) with spinal cord tissue samples than plasma (five metabolites). Thus, we propose using CSF samples for further spinal cord injury studies. Interestingly, four differentially expressed metabolites, including uric acid, phosphorycholine, pyridoxine, and guanidoacetic acid, were shared among CSF, plasma, and spinal cord. Uric acid, a metabolite that has been shown to have a protective effect on neuronal cells, was augmented in the injured spinal cord (Fukae et al. 2017; Sautin and Johnson 2008). However, levels of uric acid showed a decreased trend in both plasma and CSF, which is likely due to the fact that uric acid is more demanded in the injured spinal cord. Phosphorycholine is an intermediate in phospholipid biosynthesis (Gibson and Rees 2018), and its decrease suggests a membrane repair process is carried out following SCI. Consistent with observations in previous studies (Wu et al. 2016; Peng et al. 2014), we discovered that phosphorycholine levels were decreased in SCI. In addition, we also discovered that pyridoxine demonstrated a significant decrease across all injured groups. To date, its role in SCI has not been reported. In addition, levels of guanidoacetic acid were found to be significantly increased across all spinal cord injury samples. Furthermore, 4-acetamidobutanoate was found to be consistently higher in tissue samples from all SCI groups. Both guanidinoacetate and 4-acetamidobutanoate are downstream metabolites of arginine, which indicates that arginine metabolism plays a crucial role in SCI, which is consistent with previous report using the CSF (Wu et al. 2016). As these four metabolites were found to be differently altered across all sample types, they are worth being further studied for improved understanding of the pathophysiological process of SCI. Additionally, within the CSF and plasma, levels of phosphorylcholine and pyridoxine were demonstrated to be associated with injury-severity, suggesting that these metabolites can be utilized for assessment of different injury severities.

The subsequent pathway analysis revealed that plasma and CSF demonstrate similar pathways in response to the SCI. These pathways are highly associated with amnio acid metabolism, including phenylalanine and arginine metabolism, which was reported in the CSF of SCI patients (Wu et al. 2016). As only one hit, linoleic acid was significantly altered in the linoleic acid metabolism pathway and was associated with SCI. Herein, we observed about a twofold increase of linoleic acid in the middle and heavy SCI tissue sample. This is especially important as it has been reported in literature that linoleic acid plays a protective role in neurological system by reducing inflammation within the CNS (Monaco et al. 2018; Kong et al. 2019).

In summary, compared to plasma, metabolic profiling of CSF more accurately reflects metabolic changes within the spinal cord tissue. Four differential metabolites were commonly discovered across all three sample types. Among them, phosphorylcholine and pyridoxine showed potential application in severity assessment. However, the establishment of biomarkers for SCI requires validation across larger spinal scale studies. Ultimately, we highlighted that these neurochemical biomarkers that we identified have the potential to facilitate validation of novel therapies, and critical for translational research in SCI.

## Conclusion

In summary, we explored metabolome alterations in CSF, plasma, and spinal cord tissue after SCI in this study. Metabolite levels of phosphorycholine, pyridoxine, and guanidoacetic acid were significantly altered within the CSF, plasma, and spinal cord tissue of the spinal cord injured rats. Furthermore, levels of these three metabolites were associated with severity of SCI, suggesting that they may be potential biomarkers for the prognosis of SCI.

## Materials and Methods

### Study Design

We designed the study based on the workflow of UHPLC-HRMS-based untargeted metabolomic profiling of CSF, plasma, and spinal cord tissue samples from control and spinal cord injury rats (Fig. 1).

### Animals and Chemicals

All experiments were granted approval by the Institutional Animal Care and Use Committee of Jinan University, China. The Experimental Animal Center of Sun Yat-sen University provided female Sprague–Dawley rats (180–220 g body weight). These 24 rats were classified randomly into four groups (6 rats per group), including sham (laminectomy only), light injury, moderate injury, and heavy injury groups (spinal cord injury induction at the T10 spinal segment). These rats were placed in individual cages at 25 ± 3 °C, with open access to water and food.

Fisher (Fisher Chemical, Germany) provided the MS-grade acetonitrile, formic acid, methanol, and water. Traceless ammonium formate was bought from Sigma Chemical (St. Louis, USA). Millipore-Q water purification system (Bedford, USA) was utilized to produce ultra-high purity water. The 2-chlorobenzalanine was acquired from TCI chemicals.

### Spinal Cord Injury Rat Models

Established methods were followed for surgical procedures in order to induce spinal cord injury (Wu et al. 2019b). For anesthesia, rats were intraperitoneally injected with 10% chloral hydrate (0.35 mL/100 g body weight). After being anesthetized, the rats were dorsally incised to form a median longitudinal slit of 2.5 cm with T10 spinous process in the middle in order to expose the T9–11 spinous processes and laminae. The T10 lamina was then completely amputated, and an approximately 10-mm segment of the spinal cord was exposed. The T10 facets were then fixed bilaterally using a stabilizer. The pressure of the nitrogen tank that controls the impact tip was then adjusted to 18 psi or 124 kPa. The U-shaped stabilizer with the rat was loaded onto a stage of the Louisville Injury System Apparatus (LISA). The height of the dura/spinal cord was then adjusted directly under the impactor and was monitored using the laser beam. The crash depth needs to be adjusted according to different damage levels for the crash (the time is 0.5 s). The crash depth of light injury is 0.6 mm, moderate injury is 1.0 mm, and heavy injury is 1.8 mm. After injury, the U-shaped stabilizer was then detached from the stage, and the rat was removed from the stabilizer. The injury area was then examined and, if present, the bleeding was stopped. The muscles and skin were then sutured into layers using a 3–0 silk surgical suture. All animals with spinal cord injury followed the criteria of having flicking movement in the legs and body, as well as tail sway reflex formation, ischemia of the spinal cord, and edema covering the wound and appearing sluggishly paralyzed. The T10 total laminectomy was carried out in the sham group of rats in order to keep the spinal cord intact. These rats were put into cages with provisions of adequate food and water. In addition, an injection of gentamicin injection at a dose of 2,000 U/day was administered intraperitoneally for their treatment. The bladder was manually pressed every 8 h in order to assist in urination until spontaneous voiding recurred.

### Preparation of the Samples for LC–MS Analysis

The plasma and CSF samples were collected according to methods that have been previously described (Verwaest et al. 2011; Wu and Feng 2016). In brief, at the endpoint of the study, the rats were anesthetized, and blood was sampled from the hepatic portal vein into the heparin anticoagulation tubes. It was then immediately centrifuged at 2500 × g (10 min, 4 °C). For CSF samples, after rat anesthesia, the rats’ head were bent downward at nearly 45°. The CSF collection was carried out by puncturing the cisterna magna with a 23 G needle, with no incision within the region. Approximately 200 μL sample was taken into the syringe using simple aspiration. The process of metabolite extraction was as follows. Frozen samples were thawed on ice, and four times volume of chilled methanol was added to 100 μL plasma (or CSF). The solution was mixed rigorously on a vortex for 60 s and placed into tubes at 4 °C for 20 min. After, centrifugation of all samples at 12,000 rpm at 4 °C for 10 min was performed. Next, the supernatant was poured into a new centrifuge tube and desiccated by a speed vacuum. The dried samples were then reconstituted into 150 μL of 80% methanol containing 4 ppm 2-chlorobenzalanine. For spinal cord tissue samples, an equivalent amount (100 mg) of spinal cord tissue was placed into pre-cooled homogenization tubes to contain five steel balls and 1 ml ice-cold 80% methanol. Next, homogenization of samples was carried out at a rate of 70 Hz for 60 s by using high flux organization grinding apparatus. Metabolites were further extracted through the use of an ultrasonic machine at room temperature, for 30 min, and subsequently, centrifugation was performed at 12,000 rpm for 10 min at a temperature of 4 °C. Equal volume (800 μL) of the supernatant was poured into a fresh centrifuge tube and desiccated by a speed-vacuum. The extracts were reconstituted in 400 μL of 50% methanol (containing 4 ppm 2-chlorobenzalanine) and filtered via a 0.22-μm membrane. In order to monitor the signal response of LC–MS instrument, a pooled quality control (QC) sample was developed by blending 20 μL of each sample, which was then repeatedly injected. All samples were preserved at −80 °C unless LC–MS analysis was performed.

### LC–MS Analysis and Data Processing

Chromatographic separation was conducted on a Dionex Ultimate 3000 system that is endowed with a binary pump, a heated column oven, and a WPS-3000 autosampler. The autosampler temperature was then fixed at 8 °C. Next, 2 μL of each sample was loaded on an ACQUITY UPLC® HSS T3 (150 × 2.1 mm, 1.8 µm, Waters) column retained at a temperature of 40 °C at a flow rate of 0.2 mL/min. A mixture of 5 mM ammonium formate and 0.1% formic acid in the water forms mobile phase A. Similarly, a mixture of 0.1% formic acid and acetonitrile forms mobile phase B. Likewise, 0.1% of formic acid was mixed in water in order to form mobile phase C. However, a mixture of 0.1% formic acid and acetonitrile forms mobile phase D. In a positive mode, a linear gradient was used next: 0 ~ 1 min, 2% D; 1 ~ 9 min, 2% ~ 50% D; 9 ~ 12 min, 50% ~ 98% D; 12 ~ 13.5 min, 98% D; 13.5 ~ 14 min, 98% ~ 2% D; 14 ~ 20 min, 2% D. The gradient that was used in the negative mode was as follows: 0 ~ 1 min, 2% B; 1 ~ 9 min, 2% ~ 50% B; 9 ~ 12 min, 50% ~ 98% B; 12 ~ 13.5 min, 98% B; 13.5 ~ 14 min, 98% ~ 2% B; 14 ~ 20 min, 2% B. The MS acquisition was carried out on a Q-Exactive orbitrap MS. The ESI source parameters were fixed as follows. The spray voltage in negative mode was set at −2.5 kV, while in the positive mode, it was set at 3.8 kV. The auxiliary gas and sheath gas were set at 10 and 30 arbitrary units, while the capillary temperature was kept at 325 °C. The orbitrap mass analyzer scanned over a mass range of *m/z* 80–1000 in full scan mode at a mass resolution of 70,000. In addition, data-dependent acquisition (DDA) MS/MS experiments were carried out using normalized collision energy 30 in order to obtain the ms/ms spectra of metabolites-of-interest. The metabolite identification was achieved by matching the acquired MS/MS spectra with the reference spectra in HMDB and MassBank.

### Data Processing and Statistical Analysis

All the raw LC–MS files were changed to the mzxml format using Proteowizad software (Chambers et al. 2012), and subsequently processed using the XCMS package for peak picking, retention time alignment, and grouping (Smith et al. 2006). The major parameters of XCMS included: bw = 5, ppm = 15, peak width = c(10,20), mzdiff = 0.01, and method = centWave. The three-dimensional datasets containing the *m/z*, retention time (*rt*), and peak intensity information of each sample were then exported for further analysis.

The obtained data matrix was then manually inspected in Microsoft Excel (Microsoft Office 2010). Statistical analysis was conducted by retaining only such LC–MS features, as shared by more than 50% of samples (Cuevas-Delgado et al. 2020). The peak intensity of individual LC–MS feature in each sample was normalized to peak intensity of total useful signal (Cuevas-Delgado et al. 2020). Multivariate (PCA and PLS-DA) and univariate (ANOVA) statistical analyses were carried out by the metaboanalyst (www.metaboanalyst.ca) platform (Chong et al. 2019). Prior to the PCA and PLS-DA analysis, data was further mean-centered and auto-scaled. For the univariate analysis, one-way ANOVA with a Tukey HSD post hoc test was used to compare the means between the multiple groups; a *P*-value < 0.05 is considered as statistically significant.

## Data Availability

The datasets generated and analyzed during the current study will be publicly available after the manuscript is accepted.

## Declarations

### Disclaimer

The funders had no role in the design of the study; in the collection, analyses, or interpretation of data; in the writing of the manuscript, or in the decision to publish the results.

## Abbreviations

CSF: Cerebrospinal fluid
LC-MS: Liquid chromatogram-mass spectrometry
PCA: Principal component analysis
PLS-DA: Partial least squares discriminant analysis
ANOVA: Analysis of variance
L: Light injury
M: Moderate injury
H: Heavy injury
